# Cardiovascular outcomes between dapagliflozin versus empagliflozin in patients with diabetes mellitus

**DOI:** 10.1002/clc.24248

**Published:** 2024-03-04

**Authors:** Jee‐Heon Kim, Young‐Chae Yoon, Young‐Hoon Kim, Jong‐Il Park, Kang‐Un Choi, Jong‐Ho Nam, Chan‐Hee Lee, Jang‐Won Son, Jong‐Seon Park, Ung Kim

**Affiliations:** ^1^ Yeungnam University College of Medicine Daegu Republic of Korea; ^2^ Division of Cardiology Yeungnam University Medical Center Daegu Republic of Korea

**Keywords:** dapagliflozin, diabetes mellitus, empagliflozin, major adverse cardiovascular events

## Abstract

**Background:**

Sodium‐glucose co‐transporter 2 (SGLT2) inhibitors have been demonstrated to decrease cardiovascular adverse events. However, there is little real‐world clinical evidence regarding a direct comparison between dapagliflozin and empagliflozin in patients with diabetes mellitus (DM).

**Hypothesis:**

A difference in the cardiovascular efficancy of dapagliflozin versus empagliflozin in DM patients was anticipated, aiming to guide the optimal choice of SGLT2 inhibitors based on cardiovascular outcomes.

**Methods:**

From 2014 to 2020, a total of 1549 patients with DM who were prescribed SGLT2 inhibitors such as dapagliflozin or empagliflozin were retrospectively enrolled. We categorized the study population into two groups: dapagliflozin (*n* = 981) and empagliflozin group (*n* = 568). The primary endpoint was major adverse cardiovascular events (MACE), defined as a composite of all‐cause death, myocardial infarction (MI), stroke, or hospitalization for heart failure (HF) over a 3‐year period.

**Results:**

Propensity‐score matching was performed (537 patients in each group). The mean age and hemoglobin A1c were 58.2 ± 13.0 years and 8.4 ± 1.7%, respectively. There was no significant difference between the dapagliflozin and empagliflozin groups in the risk of MACE (3.7% vs. 4.8%, hazard ratio [HR], 1.31; 95% confidence interval [CI], 0.73–2.35; *p* = 0.349). Furthermore, there were no differences between the two groups in secondary endpoints including all‐cause death, MI, stroke, and hospitalization for HF. Prior MI and history of HF were independent predictors of MACE.

**Conclusions:**

Dapagliflozin and empagliflozin showed no significant difference of real‐world clinical cardiovascular outcomes in patients with DM over a 3‐year period. Further large randomized clinical trials will be warranted for better evaluation.

## Graphical abstract

Comparative study on dapagliflozin and empagliflozin showed similar 3‐year cardiovascular outcomes in patients with diabetes mellitus. DM, diabetes mellitus; MACE, major adverse cardiovascular events; PS, propensity‐score; SGLT2, sodium‐glucose co‐transporter 2.

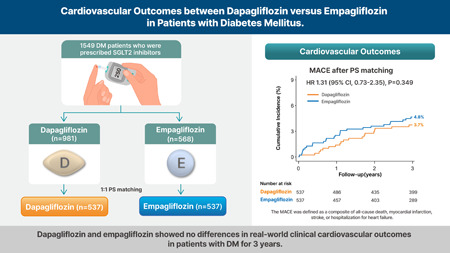

**Abbreviations**
ACE: angiotensin‐converting enzyme
ARB: angiotensin receptor blocker
DM: diabetes mellitus
HF: heart failure
MACE: major adverse cardiovascular events
MI: myocardial infarction
PCI: percutaneous coronary intervention
PS: propensity score
SGLT2: sodium‐glucose co‐transporter 2

## 1 INTRODUCTION

Diabetes mellitus (DM) is a major public health concern globally, known for its association with a spectrum of complications, including atherosclerotic cardiovascular disease and heart failure.^1^, ^2^ Effective management of blood glucose levels is pivotal in mitigating these risks. Sodium‐glucose co‐transporter 2 (SGLT2) inhibitors, such as dapagliflozin and empagliflozin, have emerged as prominent agents in this context. These inhibitors offer several advantages over traditional glucose‐lowering agents, including weight loss and blood pressure reduction without the risk of hypoglycemia.^3^, ^4^ Crucially, SGLT2 inhibitors achieve glycemic control independently of insulin action, functioning by inhibiting glucose reabsorption in the kidneys and promoting urinary glucose excretion.^5^ What sets dapagliflozin and empagliflozin apart in the realm of DM management is their documented cardiovascular benefits. Several clinical trials have highlighted their efficacy in reducing cardiovascular risks in patients with DM, as well as in those suffering from heart failure with reduced ejection fraction.^6^, ^7^, ^8^, ^9^ Their benefits extend even to patients with heart failure with preserved ejection fraction, as recent studies have shown.^10^, ^11^ In light of these findings, current guidelines now recommend to use of SGLT2 inhibitors for heart failure patients, irrespective of their ejection fraction status.^12^ Despite these advances, there exists a notable gap in real‐world data, particularly concerning direct comparison between dapagliflozin and empagliflozin in patients with DM. This is especially true in global contexts where these medications are widely prescribed. Addressing this gap is essential for informed clinical decision‐making and optimizing patient outcomes. Therefore, our study aimed to fill this void by providing a direct, real‐world comparison of the cardiovascular protective effects of dapagliflozin and empagliflozin in patients with DM. Through this research, we seek to contribute valuable insights to the existing literature and aid clinicians in making more informed treatment choices for their patients with DM.

## 2 METHODS

### 2.1 Study population and data collection

**Figure 1:**
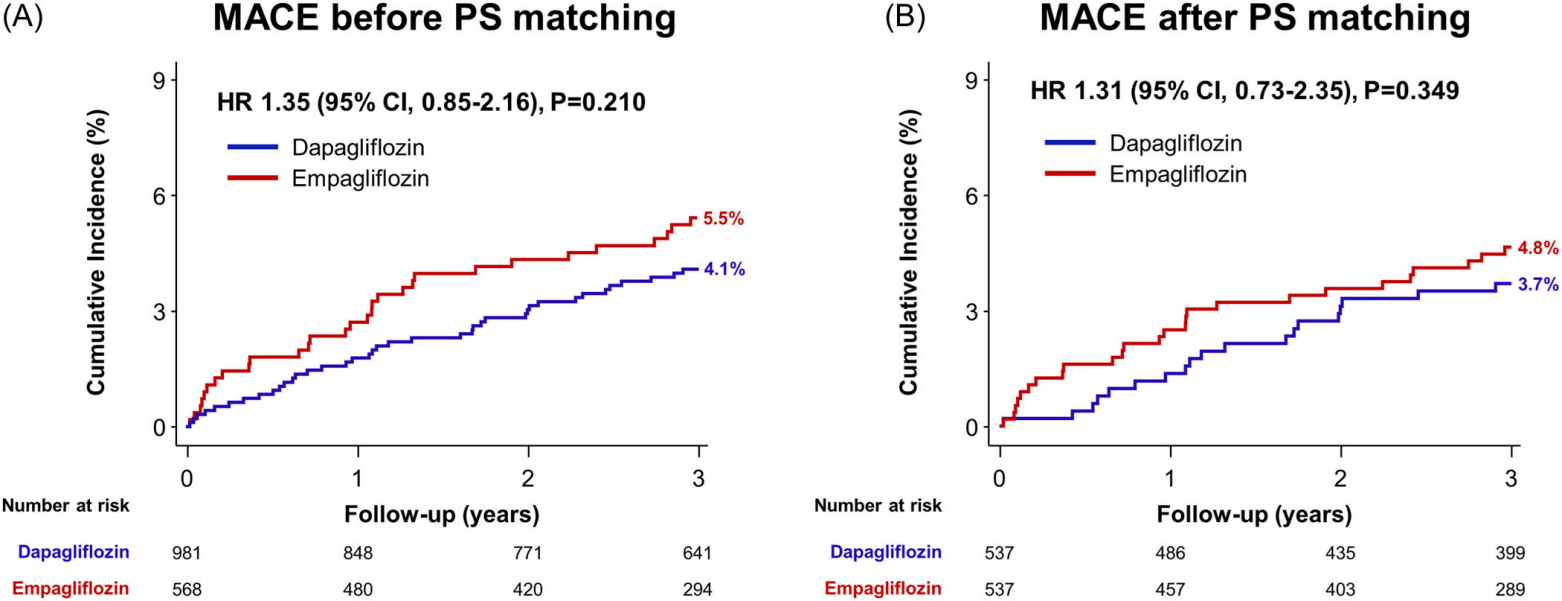
Time‐to‐event curves of the primary endpoint according to the types of SGLT2 inhibitor (A) in the total population, (B) in the propensity score‐matched population. Time‐to‐event curves were plotted using Kaplan–Meier survival analysis. CI, confidence interval; HR, hazard ratio; MACE, patient oriented composite endpoint; PS, propensity score.

This study was a retrospective observational study that enrolled 2212 diabetic patients prescribed dapagliflozin or empagliflozin from November 2014 to September 2020 at Yeungnam University Medical Center. For this study, 663 patients without follow‐up data were excluded. A total of 1549 patients were included in the final analysis. (Supporting Information S3: Figure 1). The study population was stratified into two groups with respect to prescribed SGLT2 inhibitor: dapagliflozin (*n* = 981) and empagliflozin (*n* = 568). Data on baseline medial history, medications, procedural records, and 3‐year clinical outcomes were collected from the patients' electronic medical records or direct telephone contact.

### 2.2 Study endpoints and definitions

The primary endpoint of this study was 3‐year major adverse cardiovascular events (MACE). MACE was defined as a composite of all‐cause death, myocardial infarction, stroke, or hospitalization for heart failure. The secondary endpoints included individual components of the primary endpoint and hospitalization for ischemic heart disease. Myocardial infarction (MI) was defined as cardiac biomarker elevation with at least one value above the 99th percentile of the upper reference limit, with accompanying ischemic symptoms or electrocardiographic findings indicative of ischemia unrelated to an intervention procedure.^13^ Stroke was defined as an acute cerebrovascular event resulting in a neurologic deficit >24 h or the presence of acute infarction demonstrated by imaging studies.^14^ Hospitalization for heart failure was defined as hospitalization requiring at least an overnight stay due to substantial worsening of heart failure symptoms and/or signs, necessitating he augmentation of oral medications or new administration of intravenous heart failure therapy, including diuretics, inotropes, or vasodilators.^15^ Hospitalization for ischemic heart disease was defined as hospitalization due to the need for coronary revascularization for typical symptoms and signs of electrocardiographic changes, exercise, or pharmacological stress, study evidence for inducible myocardial ischemia, angiographic evidence for new or worsening coronary artery disease and/or intracoronary thrombus, or a hospitalization requiring at least an overnight stay due to substantial worsening of ischemic symptoms and signs (electrocardiographic, echocardiographic, or biomarker changes).^16^ Chronic kidney disease was defined as an eGFR <60 mL/min/1.73 m^2^, including end‐stage renal disease with renal replacement therapy.

### 2.3 Statistical analysis

Baseline patient characteristics were tested using Student's *t* test for continuous variables and *χ* ^2^ statistics for categorical variables. To minimize the impact of differences in baseline characteristics between two patient groups, the propensity score (PS) matching method was conducted. PS were obtained from logistic regression, including variables that exhibited a significant difference between the two groups (sex, prior MI, prior stroke, hypertension, dyslipidemia, history of heart failure, beta‐blocker, ARB/ACE inhibitor, and aspirin use). The Cox proportional hazards regression model was used to calculate the hazard ratio for the primary endpoint and secondary endpoints. In univariate analysis, a Cox proportional‐hazards regression model was conducted for several clinical variables, and the variables achieving *p* < 0.05 in the univariate analysis were entered into the multivariate analysis model to determine independent predictors of MACE. Event‐free survival was analyzed using Kaplan–Meier survival curves, and differences between event‐free survival curves were compared using the log‐rank test. All hazard ratios were calculated with a 95% CI and *p *< 0.05 was considered statistically significant. The collected data were stored in Excel spreadsheets and statistical analyses were performed using SPSS version 27.0.0 (IBM).

## 3 RESULTS

### 3.1 Baseline characteristics

The mean age of the patients was 58.2 ± 13.0 years, and the average hemoglobin A1c level was 8.4 ± 1.7%. The mean follow‐up duration was 1227.4 ± 603.4 days. While the mean age was comparable between the two groups (58.3 ± 13.4 vs. 58.0 ± 12.3 years, *p* = 0.697), significant differences were noted in sex distribution and medical histories (Table 1). The empagliflozin group had a higher proportion of males (59.7% vs. 65.8%, *p* = 0.017) and a greater prevalence of prior MI (9.9% vs. 15.1%, *p* = 0.002), acute coronary syndrome (13.1% vs. 17.1%, *p* = 0.037), prior stroke (11.6% vs. 19.2%, *p* < 0.001), dyslipidemia (43.9% vs. 52.1%, *p* = 0.002), history of heart failure (11.7% vs. 16.7%, *p* = 0.007), current smoker (11.1% vs. 18.1%, *p* < 0.001), and usage of specific cardiovascular medications. After applying PS matching to balance these baseline characteristics, each group consisted of 537 patients, making a total of 1074 patients. This matching process effectively equalized the baseline differences, making the groups more comparable for outcome analysis.

**Table 1:** Baseline characteristics.

	Before propensity score matching (*N* = 1549)			After propensity score matching (*N* = 1074)		
Characteristics	Dapagliflozin (*N* = 981)	Empagliflozin (*N* = 568)	*p* Value	Dapagliflozin (*N* = 537)	Empagliflozin (*N* = 537)	*p* Value
Age, years	58.3 ± 13.4	58.0 ± 12.3	0.697	57.0 ± 13.1	57.7 ± 12.4	0.368
Male, sex	586 (59.7)	374 (65.8)	0.017	350 (65.2)	353 (65.7)	0.898
Hypertension	484 (49.3)	329 (57.9)	0.001	304 (56.6)	306 (57.0)	0.951
Dyslipidemia	431 (43.9)	296 (52.1)	0.002	301 (56.1)	273 (50.8)	0.099
Chronic kidney disease^a^	144 (14.7)	95 (16.7)	0.307	84 (15.6)	90 (16.8)	0.679
Acute coronary syndrome^b^	129 (13.1)	97 (17.1)	0.037	98 (18.2)	85 (15.8)	0.330
Prior PCI	136 (13.9)	93 (16.4)	0.182	96 (17.9)	82 (15.3)	0.286
Prior myocardial infarction	97 (9.9)	86 (15.1)	0.002	71 (13.2)	74 (13.8)	0.858
Prior stroke	114 (11.6)	109 (19.2)	<0.001	93 (17.3)	94 (17.5)	1.000
History of heart failure	115 (11.7)	95 (16.7)	0.007	84 (15.6)	80 (14.9)	0.799
Serum hemoglobin, mg/dL	14.0 ± 2.0	13.8 ± 1.9	0.208	14.1 ± 1.8	13.9 ± 2.0	0.084
Hemoglobin A1c, %	8.3 ± 1.7	8.5 ± 1.7	0.079	8.4 ± 1.7	8.5 ± 1.7	0.373
Smoking status						
Never smoker	665 (67.8)	338 (59.5)	<0.001	352 (65.5)	330 (61.5)	0.183
Former smoker	207 (21.1)	127 (22.4)	0.565	105 (19.6)	124 (23.1)	0.180
Current smoker	109 (11.1)	103 (18.1)	<0.001	80 (14.9)	83 (15.5)	0.432
Medications						
Beta‐blocker	201 (20.5)	159 (28.0)	0.001	138 (25.7)	135 (25.1)	0.889
ARB/ACE inhibitor	377 (38.4)	253 (44.5)	0.021	233 (43.4)	232 (43.2)	0.999
Calcium channel blocker	187 (19.1)	115 (20.2)	0.595	120 (22.3)	109 (20.3)	0.456
Mineralocorticoid	33 (3.4)	28 (4.9)	0.137	17 (3.2)	23 (4.3)	0.421
Aspirin use	244 (24.9)	211 (37.1)	<0.001	166 (30.9)	193 (35.9)	0.093

*Note*: Data are presented as mean ± SD, median (interquartile range), or number (%).

Abbreviations: ACE inhibitor, angiotensin‐converting‐enzyme inhibitor; ARB, angiotensin receptor blocker; PCI, percutaneous coronary intervention.

^a^ Chronic kidney disease was defined as an estimated glomerular filtration rate of <60 mL/min/1.73 m^2^ of body surface area.

^b^ Acute coronary syndrome included non‐ST‐elevation myocardial infarction, ST‐elevation myocardial infarction, and unstable angina.

### 3.2 Three‐year clinical cardiovascular outcomes

**Figure 2:**
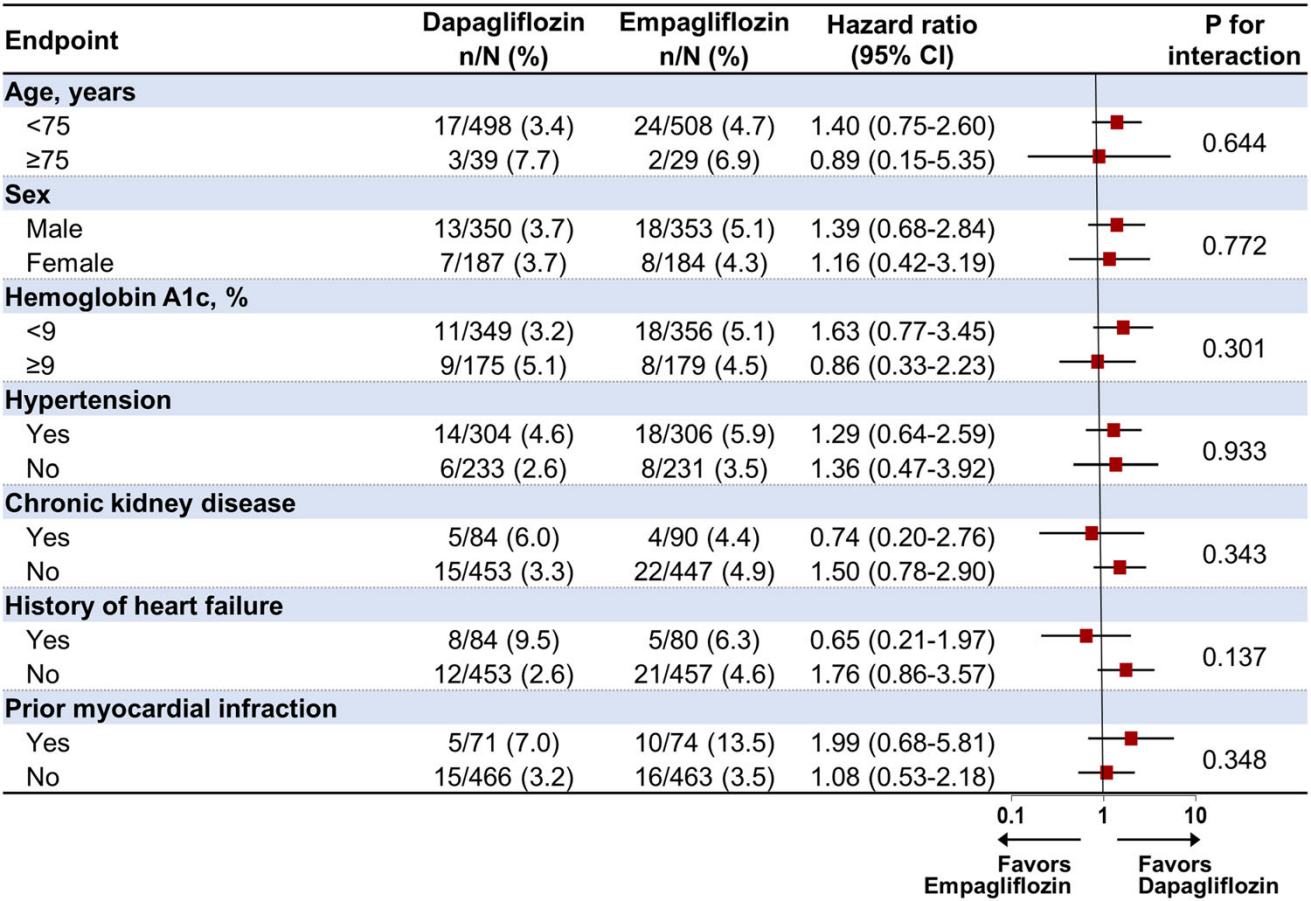
Subgroup analysis for the primary endpoint of the propensity score‐matched population. CI, confidence interval.

The overall incidence of the primary endpoint, MACE, was 4.6% in the total study population. Before PS matching, MACE occurred in 4.1% of the dapagliflozin group and 5.5% of the empagliflozin group (hazard ratio [HR], 1.35; 95% CI, 0.85–2.16; *p* = 0.210). Post PS matching analysis also showed no significant difference in MACE between the two groups (3.7% in dapagliflozin vs. 4.8% in empagliflozin; HR, 1.31; 95% CI, 0.73–2.35; *p* = 0.349) (Figure 1, Table 2). Similarly, no significant differences were observed in hospitalization for heart failure (1.7% vs. 3.4%; HR, 2.02; 95% CI, 0.91–4.49; *p* = 0.085) and other secondary endpoints, including hospitalization for ischemic heart disease (Supporting Information S3: Figure 2, Table 2).

**Table 2:** Three‐year clinical efficacy endpoint according to SGLT2 inhibitors before or after propensity score matching.

	**Before PSM (*N*** = **1549)**				**After PSM (*N*** = **1074)**			
	**Dapagliflozin (*N*** = **981)**	**Empagliflozin (*N*** = **568)**	**Adjusted HR (95% CI)**	***p* Value**	**Dapagliflozin (*N*** = **537)**	**Empagliflozin (*N*** = **537)**	**Adjusted HR (95% CI)**	***p* Value**
Primary endpoint (MACE)	40 (4.1)	31 (5.5)	1.35 (0.85–2.16)	0.210	20 (3.7)	26 (4.8)	1.31 (0.73–2.35)	0.349
Composite of all cause death, myocardial infarction, stroke, or hospitalization for heart failure								
Individual clinical endpoints								
All cause death	9 (0.9)	7 (1.2)	1.35 (0.50–3.61)	0.556	4 (0.7)	6 (1.1)	1.50 (0.42–5.33)	0.528
Cardiovascular death	4 (0.4)	5 (0.9)	2.16 (0.58–8.05)	0.250	2 (0.4)	4 (0.7)	2.01 (0.37–10.95)	0.422
Noncardiovascular death	5 (0.5)	2 (0.4)	0.69 (0.13–3.57)	0.659	2 (0.4)	2 (0.4)	1.00 (0.14–7.11)	0.999
Myocardial infarction	15 (1.5)	14 (2.5)	1.63 (0.79–3.37)	0.191	8 (1.5)	12 (2.2)	1.51 (0.62–3.70)	0.366
Stroke	13 (1.3)	4 (0.7)	0.80 (0.38–1.67)	0.553	7 (1.3)	3 (0.6)	0.23 (0.11–1.66)	0.221
Hospitalization for heart failure	21 (2.1)	22 (3.9)	1.74 (0.95–3.19)	0.073	9 (1.7)	18 (3.4)	2.02 (0.91–4.49)	0.085
Hospitalization for ischemic heart disease	25 (2.5)	19 (3.3)	1.33 (0.73–2.41)	0.352	16 (3.0)	17 (3.2)	1.07 (0.54–2.12)	0.845

*Note*: Data are the number of events percentages. Hazard ratios were calculated by Cox regression analysis in which age, sex, acute coronary syndrome, prior stroke, and dyslipidemia were included for adjustment.

Abbreviations: CI, confidence interval; PSM, propensity score match.

### 3.3 Subgroup analysis

Figure 2 presents a forest plot illustrating the comparison of the primary endpoint between dapagliflozin and empagliflozin across various subgroups, including age (<75 and ≥75 years), sex, hemoglobin A1c level (<9 and ≥9%), hypertension, chronic kidney disease, history of heart failure, and prior MI. No significant differences were observed in the subgroup analysis of the primary endpoint between dapagliflozin and empagliflozin.

### 3.4 Independent predictors of the primary endpoint

In the total study population, several clinical variables were significantly associated with the prevalence of MACE (Table 3). These included age ≥75 years (HR, 2.21; 95% CI, 1.09–4.14; *p* = 0.027), hypertension (HR, 1.91; 95% CI, 1.16–3.14; *p* = 0.011), prior MI (HR, 3.22; 95% CI, 1.93–5.36; *p* < 0.001), a history of heart failure (HR, 2.58; 95% CI, 1.54–4.33; *p* < 0.001). Multivariate analysis further identified prior MI (HR, 2.62; 95% CI, 1.61–4.52; *p* = 0.001) and history of heart failure (HR, 1.91; 95% CI, 1.10–3.33; *p* = 0.023) as independent predictors of MACE.

**Table 3:** Independent predictors of primary endpoint in total population.

Factor	Univariate analysis		Multivariate analysis	
	HR (95% CI)	*p* Value	HR (95% CI)	*p* Value
Age ≥75 (years)	2.12 (1.09–4.14)	0.027	1.61 (0.81–3.22)	0.178
Male	1.29 (0.78–2.12)	0.316		
Hypertension	1.91 (1.16–3.14)	0.011	1.41 (0.83–2.38)	0.205
Dyslipidemia	1.10 (0.69–1.75)	0.701		
Chronic kidney disease	1.36 (0.75–2.43)	0.308		
Prior myocardial infarction	3.22 (1.93–5.36)	<0.001	2.62 (1.61–4.52)	0.001
Prior stroke	1.46 (0.82–2.63)	0.202		
Hemoglobin A1c ≥ 9 (%)	1.13 (0.69–1.86)	0.619		
History of heart failure	2.58 (1.54–4.33)	<0.001	1.91 (1.10–3.33)	0.023
Empagliflozin	1.35 (0.85–2.16)	0.210		

Abbreviations: CI, confidence interval; HR, hazard ratio.

## 4 DISCUSSION

In this study, we demonstrated the favorable 3‐year cardiovascular outcome of SGLT2 inhibitors, with a 4.6% occurrence according to the primary endpoint in patients with DM. Additionally, there was no significant difference in the occurrence of MACE between dapagliflozin and empagliflozin. Furthermore, we found that there were no significant differences in all‐cause death, cardiovascular death, MI, hospitalization for heart failure, or stroke between the two groups. Additionally, in diabetic patients prescribed SGLT2 inhibitors, important predictors for cardiovascular outcomes were prior MI and a history of heart failure.

The SGLT2 inhibitors, including dapagliflozin and empagliflozin, have demonstrated a reduction in cardiovascular events in patients with DM. In the Dapagliflozin Effect on Cardiovascular Events‐Thrombolysis in Myocardial Infarction (DECLARE‐TIMI) 58 study, there was a 27% reduction in hospitalization for heart failure over an average study duration of 4.2 years.^6^ In the Empagliflozin Cardiovascular Outcome Event Trial in Type 2 Diabetes Mellitus Patients‐Removing Excess Glucose (EMPA‐REG OUTCOME) study, the group receiving empagliflozin showed a significant 14% reduction in the occurrence of MACE (composite of cardiovascular death, nonfatal MI, and nonfatal stroke) compared to the placebo group. Additionally, there was a notable 38% reduction in cardiovascular mortality and a significant 35% decrease in hospitalization for heart failure associated with cardiovascular events.^7^

The results from this presented study were in agreement with several studies, which found no significant differences in cardiovascular outcomes between dapagliflozin and empagliflozin.^17^, ^18^, ^19^, ^20^ These findings suggested a class of SGLT2 inhibitors in managing cardiovascular risks in DM patients, irrespective of the specific SGLT2 inhibitors used. However, contrasting outcomes have been observed in some studies. Previous meta‐analysis study revealed that empagliflozin might have a more favorable impact on mortality compared to dapagliflozin.^21^ Additionally, another meta‐analysis study also indicated that empagliflozin was more beneficial in reducing mortality and cardiovascular events.^22^ In contrast, some cohort studies showed no significant difference in overall MACE between the dapagliflozin and empagliflozin, but observed that dapagliflozin might be associated with lower risks hospitalization for heart failure.^23^, ^24^ The differences in outcomes across various studies might be attributed to differences in baseline comorbidities among the patients enrolled in these studies. As highlighted in previous meta‐analysis study, the presence of specific comorbidities in patients might influence the differential efficacy of these SGLT2 inhibitors.^25^ This suggested that patient‐specific factors, including baseline health conditions, could play a crucial role in determining the effectiveness of dapagliflozin and empagliflozin.

In a nationwide cohort study in Korea, it was shown that dapagliflozin might be more favorable in hospitalization for heart failure.^23^ Although this study enrolled a large number of patients, the proportion of the study population with MI was 2.7%, and history of heart failure was 5.0%, which is lower compared to the landmark studies like EMPA‐REG OUTCOME trial and DAPA TIMI 58 trial.^6^, ^7^, ^23^ Dapagliflozin group in DAPA TIMI 58 study showed that the rate of comorbidities of heart failure and coronary artery disease was 9.9% and 32.9%, respectively.^6^ The difference in the presence of comorbidities like MI and history of heart failure was crucial. This presented study demonstrated that independent predictors for MACE were prior MI and history of heart failure. The rate of prior MI and history of heart failure were 13.5% and 15.3% in this presented study. Our study showed that, even in data with more comorbidities such as MI and heart failure, there was no significant difference in cardiovascular outcomes over a 3‐year period between dapagliflozin and empagliflozin.

There were several limitations in this study. First, the study was a retrospective observational study based on single‐center data, which potentially subjected the findings to selection bias. PS matching was performed to mitigate known confounders and balance baseline characteristics. However, this method primarily addressed observable factors and may not have fully accounted for unmeasured variables that could have influenced clinical outcomes, leading to possible residual confounding. Second, the relatively small sample size of the study could have limited the statistical power and the number of events captured. This limitation was particularly significant in the context of rare outcomes, where larger sample sizes are typically required for detecting significant differences, potentially impacting the reliability and generalizability of the findings. Third, the study did not include information on the duration of diabetes, a critical factor influencing cardiovascular outcomes. Additionally, reliance on electronic medical records for data collection might have introduced recording biases, including possible missing data or inaccuracies. These gaps may have affected the understanding of disease progression and associated risks within the study cohort. Fourth, the timeframe of the study might not have fully reflected current heart failure treatment guidelines, such as the use of angiotensin receptor neprilysin inhibitors. Additionally, variations in treatment adherence among patients, which can significantly impact the effectiveness of medication and overall outcomes, were not accounted for in the study. These aspects were important as both changes in treatment guidelines and patient adherence could have had a significant impact of patient outcomes. Fifth, the study did not collect data on patients' lifestyle factors and dietary habits, which are significant determinants of health outcomes in diabetes and cardiovascular disease. Sixth, the variability in follow‐up duration among patients had the potential to impact individual clinical outcomes. Indeed, as this study was an observational retrospective study, it likely reflected outcomes that were more presentative of real‐world clinical practice. Given these limitations, it was evident that further studies, such as randomized controlled trials or larger multicenter observational studies, were necessary to corroborate and expand upon the findings presented in this study.

## 5 CONCLUSION

Dapagliflozin and empagliflozin showed no differences in real‐world clinical cardiovascular outcomes in patients with DM over 3 years. Further research and large randomized clinical trials may be warranted to confirm differences between SGLT2 inhibitors.

## AUTHOR CONTRIBUTIONS

*Conception and design and overall responsibility*: Jong‐Il Park and Ung Kim. *Analysis and interpretation*: Jong‐Il Park, Jee‐Heon Kim, Young‐Chae Yoon, and Young‐Hoon Kim. *Data collection*: Jee‐Heon Kim, Young‐Chae Yoon, Young‐Hoon Kim, Jong‐Il Park, Kang‐Un Choi, Jong‐Ho Nam, Chan‐Hee Lee, Jang‐Won Son, Jong‐Seon Park, Ung Kim. *Writing the article*: Jong‐Il Park, Jee‐Heon Kim, Young‐Chae Yoon, Young‐Hoon Kim. All authors contributed to the article and approved the submitted version.

## CONFLICT OF INTEREST STATEMENT

Dr. J‐I Park received funding from the 2023 Yeungnam University Research Grant. The other authors declare no conflicts of interest.

## Supporting information

Supporting information.

Supporting information.

Supporting information.

## Data Availability

The data that support the findings of this study are available from the corresponding author upon reasonable request.
