# Supplementary figures and images for: Cardiovascular outcomes between dapagliflozin versus empagliflozin in patients with diabetes mellitus
Source: Clin Cardiol. 2024 Mar 4;47(3):e24248. doi: 10.1002/clc.24248 (PMC10910463; doi:10.1002/clc.24248)

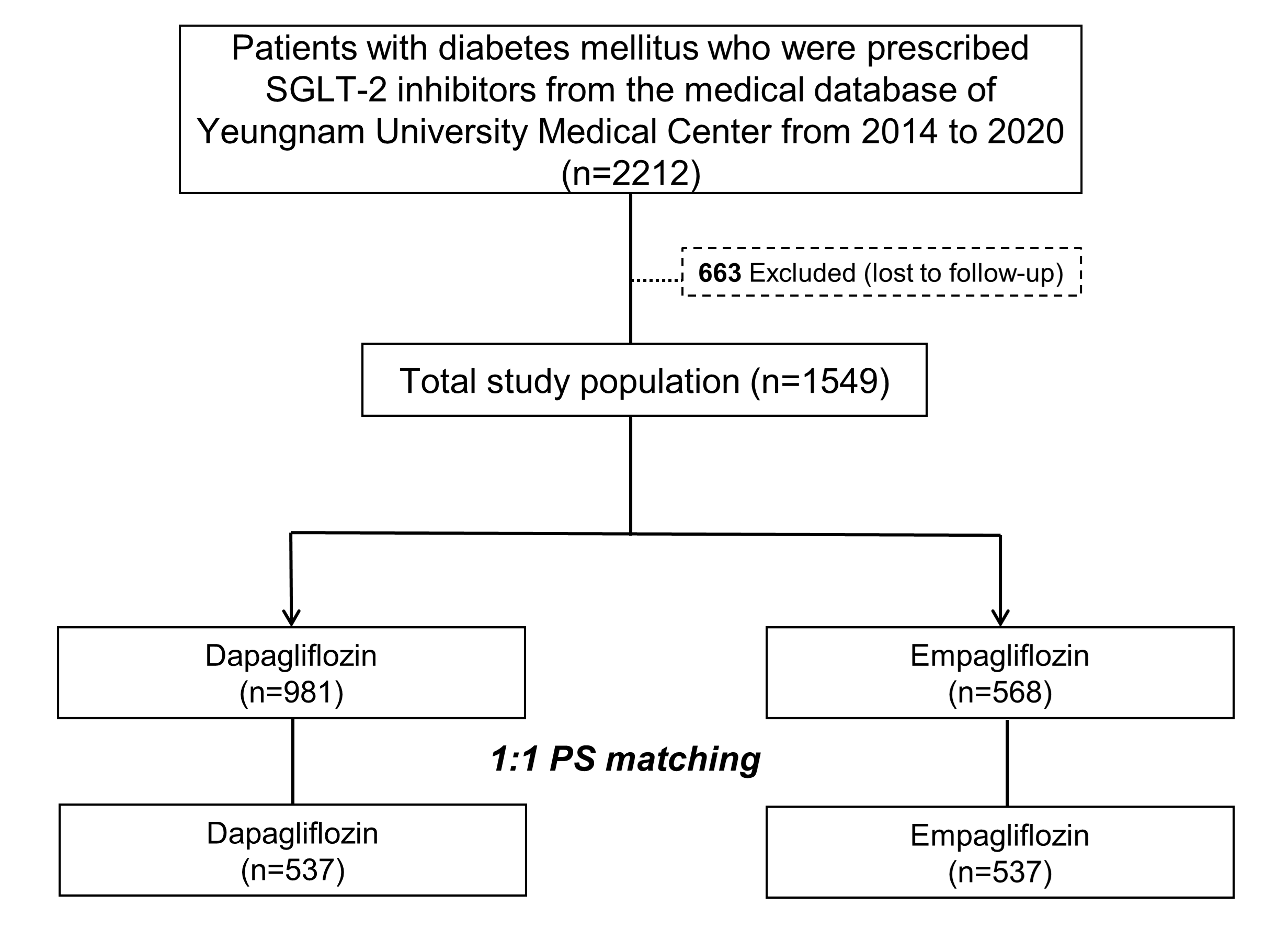

Supplement: Supplementary file 1 — Supporting information. [file CLC-47-e24248-s001.tif]

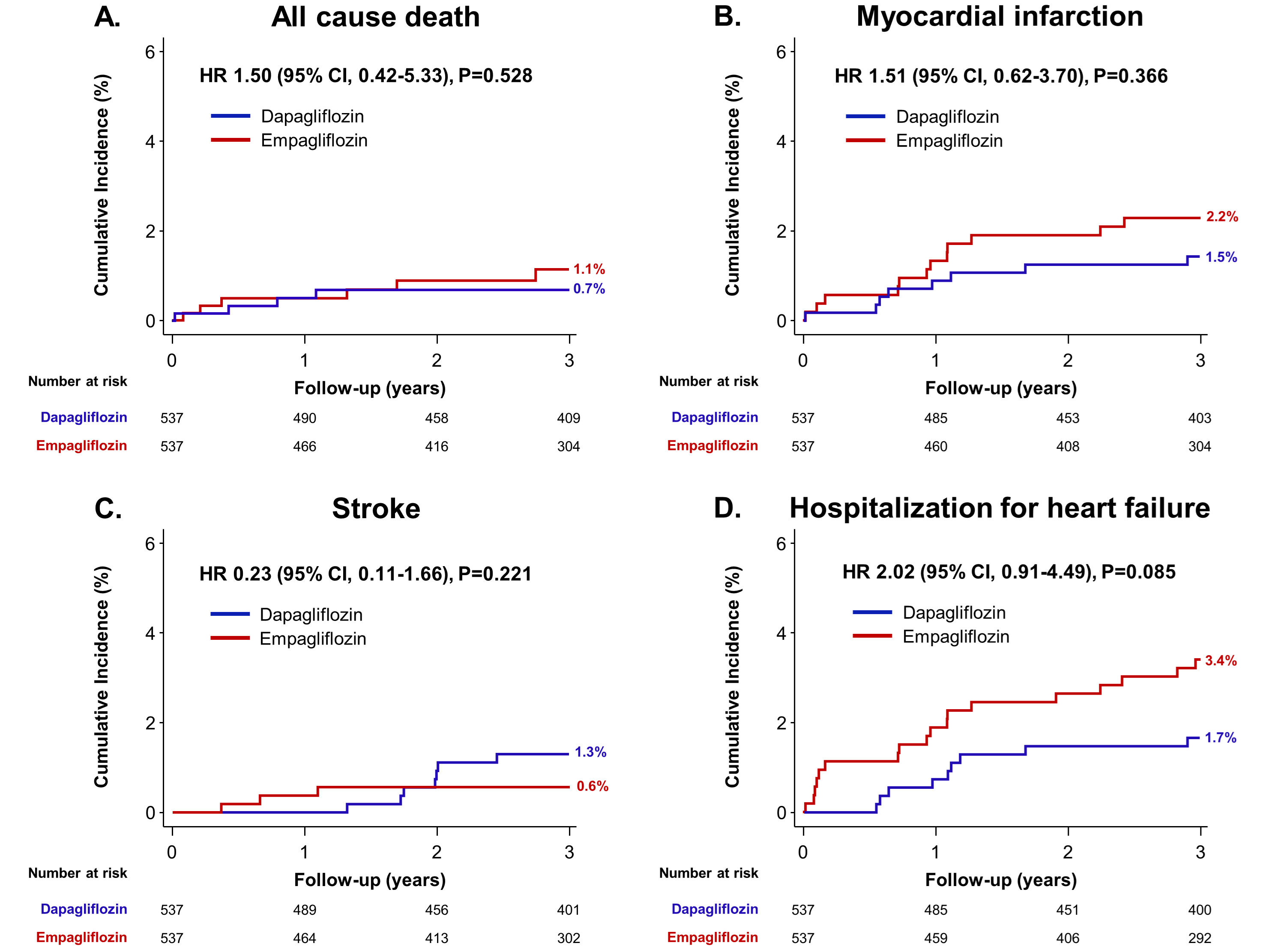

Supplement: Supplementary file 2 — Supporting information. [file CLC-47-e24248-s003.tif]
