# Supplementary material for: Cardiovascular outcomes between dapagliflozin versus empagliflozin in patients with diabetes mellitus
Source: Clin Cardiol. 2024 Mar 4;47(3):e24248. doi: 10.1002/clc.24248 (PMC10910463; doi:10.1002/clc.24248)
Supplement: Supplementary file 3 — Supporting information. [file CLC-47-e24248-s002.docx]

Supplementary material

Legends

Supplementary Figure 1. Patients flow diagram.

Supplementary Figure 2. Time-to-event curve of the secondary endpoints according to the types of SGLT2 inhibitor in the propensity score-matched population.

Supplementary Figure 1. Patients flow diagram.


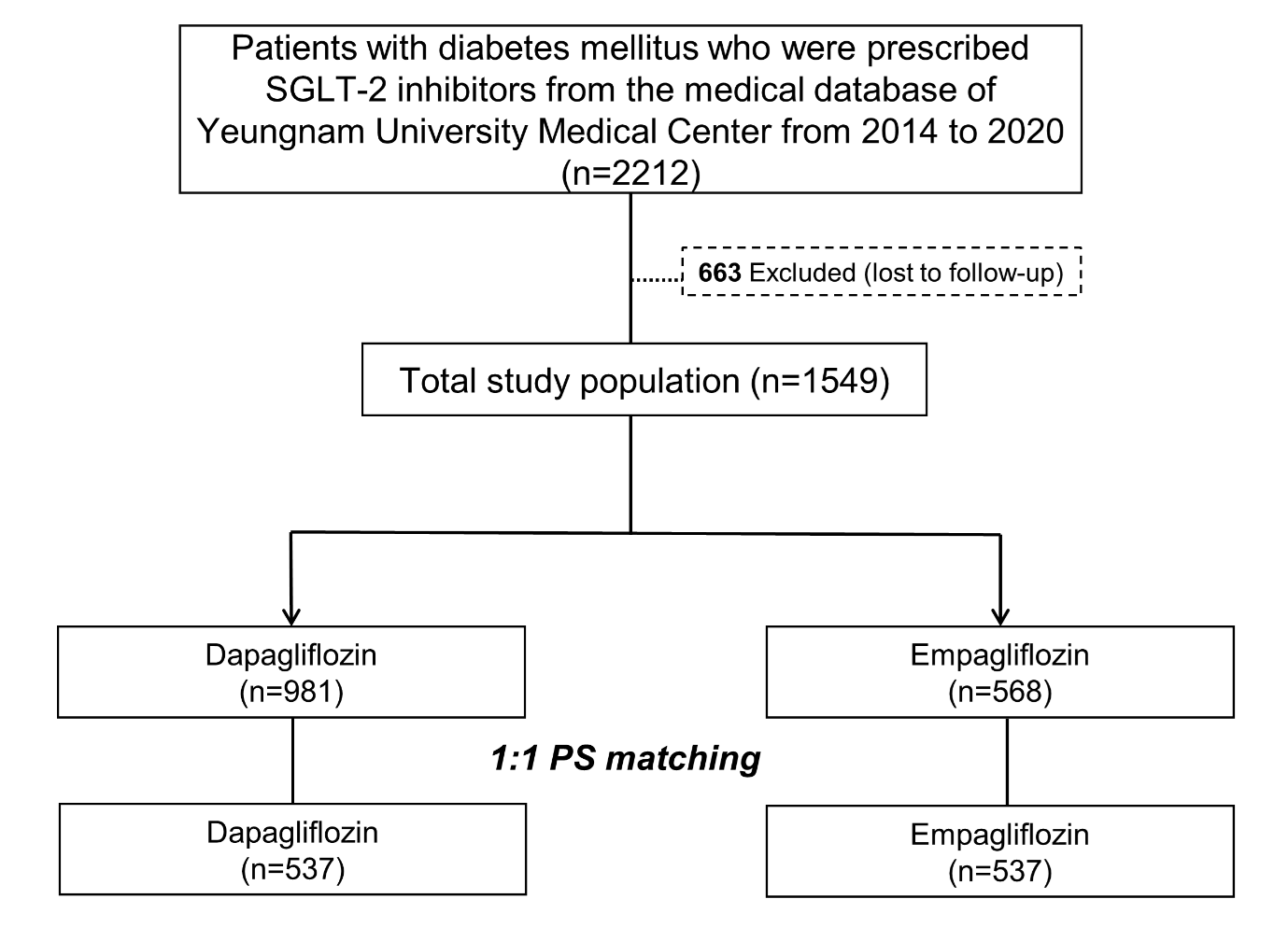


SGLT2, sodium-glucose co-transporter 2; PS, propensity score.

Supplementary Figure 2. Time-to-event curve of the secondary endpoints according to the types of SGLT2 inhibitor in the propensity score-matched population.


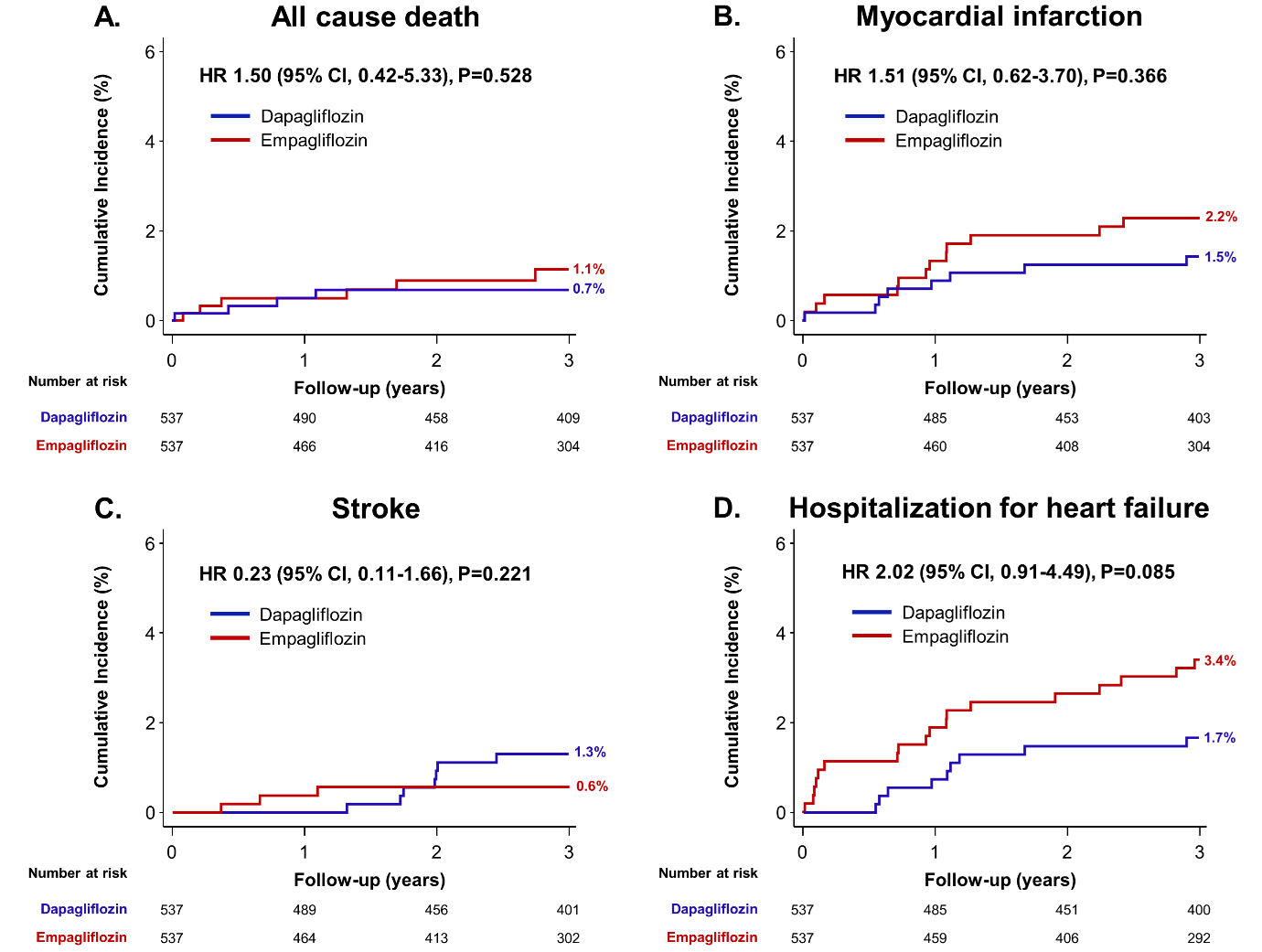


Kaplan–Meier survival curves for (A) all cause death, (B) myocardial infarction, (C) stroke, or (D) hospitalization for heart failure. HR, hazard ratio; CI, confidence interval
